# In This Issue

**Published:** 1997

## CHARTING RECENT PROGRESS: ADVANCES IN ALCOHOL RESEARCH

The *Ninth Special Report to the U.S. Congress on Alcohol and Health* is the ninth edition of a triennial report prepared by the National Institute on Alcohol Abuse and Alcoholism. This report summarizes the latest advances in alcohol-related research in a broad range of areas—including the biological, environmental, and social factors that contribute to alcohol use, abuse, and dependence; the effects of alcohol on both individuals and society; and the treatment and prevention of alcohol-related problems. This article provides an overview of the topics covered in the *Ninth Special Report* and highlights some of the recent findings revealed through the application of innovative research methods. (pp. 277–286)

## ALCOHOL-INDUCED CELL DEATH IN THE EMBRYO

In the embryo, the effects of alcohol depend on the timing of exposure and the sensitivity of a given tissue type. Studies show that alcohol profoundly affects a group of cells that are the foundation for the structures that make up the skull and face (i.e., the cranial neural crest cells). These cells are most vulnerable to alcohol’s toxic effects during a narrow window of time that occurs early in embryo development. Citing research using mice and chicks, Dr. Susan M. Smith shows that alcohol exposure during this critical window causes self-generated cell death (i.e., apoptosis) among cranial neural crest cells. Loss of these cells subsequently results in the facial defects characteristic of fetal alcohol syndrome. Dr. Smith reviews several theories on how alcohol triggers apoptosis in cranial neural crest cells. (pp. 287–295)

## NEW GENETIC TECHNOLOGIES IN ALCOHOL RESEARCH

Researchers frequently use animal models to investigate how alcohol affects certain genes and gene products and how these changes ultimately influence behavior. New techniques to generate genetically engineered animals (mostly mice) have greatly advanced scientists’ ability to analyze the effects of manipulating individual genes. These techniques include conventional and conditional gene knockout technology, in which one of the animal’s genes is inactivated, as well as conventional and regulatable transgenic technology, in which a functional foreign gene is introduced into the animal. Drs. Gregg E. Homanics and Susanne Hiller-Sturmhöfel describe these techniques and present several examples of how they may be applied to the alcohol research field. (pp. 298–309)

## SCARRING IN ALCOHOLIC LIVER DISEASE: NEW INSIGHTS AND EMERGING THERAPIES

In general, the body responds to injury by forming scars, and the liver is no different. Extensive scarring from chronic injury (e.g., because of heavy drinking) severely compromises the liver’s function, however, and eventually becomes irreversible. Given these dire consequences, researchers are actively investigating the “hows” and “whys” of scar formation on a cellular and molecular level. The goal is to find more effective therapies to reverse or prevent the damage related to widespread scarring in the liver. Dr. Scott L. Friedman reviews the latest knowledge gained from these studies—particularly new insights into the role of specialized cells in the liver known as stellate cells—and discusses several new therapeutic approaches resulting from this improved understanding. (pp. 310–316)

## CYTOKINES AND ALCOHOLIC LIVER DISEASE

Inflammation plays an important role in defending the body against infection. When inflammation persists, however, it can damage the body’s own tissues, as in the case of alcoholic liver disease. The inflammatory process is regulated in part by key hormonelike substances called cytokines. According to Drs. Craig J. McClain, Steven Shedlofsky, Shirish Barve, and Daniell B. Hill, cytokine levels rise in response to long-term alcohol consumption, leading to chronic inflammation of the liver. Subsequent changes in liver function then can cause liver failure and death. Researchers now are seeking ways to curb alcohol-induced excess cytokine activity without decreasing the body’s ability to fight infection. (pp. 317–320)

## OXIDATIVE STRESS AND ALCOHOLIC LIVER DISEASE

Most alcohol taken into the body is detoxified in the liver. Substances generated during alcohol metabolism, however, may be more toxic to the liver than alcohol itself. These substances include highly reactive molecules that can destroy vital cell components through a chemical process called oxidation. Liver cells contain natural antioxidants that help protect against excessive oxidation. Alcohol metabolism can upset the balance between oxidants and antioxidants, which, in turn, leads to cell damage and symptoms of alcoholic liver disease (ALD). Drs. Jose C. Fernández-Checa, Neil Kaplowitz, Anna Colell, and Carmen García-Ruiz explore the complex interactions between oxidants and antioxidants and discuss the potential use of antioxidants as medications to treat ALD. (pp. 321–324)

## APOPTOSIS AND NECROSIS

Alcohol-induced liver damage involves inflammation of the liver, formation of scar tissue, and the death of individual liver cells. Two mechanisms—apoptosis (or “cell suicide”) and necrosis—are believed to contribute to liver cell death. According to Drs. Amin A. Nanji and Susanne Hiller-Sturmhöfel, heavy alcohol consumption may promote apoptosis and necrosis by influencing factors that contribute to cell death, such as altering cellular metabolism and disrupting cellular communication. This article reviews the differences between these two types of cell death and speculates on their underlying mechanisms. By better understanding the mechanisms involved in cell death, researchers will be able to devise new ways of treating and preventing alcohol-related liver damage. (pp. 325–330)

## NEW MODELING METHODS

Geographic factors—such as the number of stores and bars selling alcohol and variations in the socioeconomic status of different neighorhoods—affect the patterns of alcohol use and resulting alcohol-related problems, write Dr. William F. Wieczorek and Mr. Craig E. Hanson. Computer-based geographic information systems (GIS) are now coming of age in the field of alcohol research and are enabling scientists to investigate the influence of such geographic factors in greater detail than ever before. For example, GIS can be used to readily determine the number of drinking-related crashes occurring in a certain community. Such results may play an important role in designing more effective prevention and intervention programs to reduce the incidence of these alcohol-related crashes. (pp. 331–339)

## STRATEGIES TO INCREASE ALCOHOL SCREENING IN HEALTH CARE SETTINGS

Studies have shown that simply by asking questions about alcohol use, clinicians can decrease their patients’ drinking levels. Despite the frequency with which alcohol-related problems are seen in health care settings, many clinicians still do not include screening for alcohol use disorders as part of routine patient care. Drawing from research in other prevention fields, Dr. Michael F. Fleming suggests five general strategies to encourage clinicians to provide alcohol screening for all patients. These strategies include group education (e.g., workshops or seminars), training given by respected colleagues (i.e., opinion leaders), performance feedback, educational outreach visits to individual physicians (i.e., academic detailing), and financial incentives or penalties. Dr. Fleming also suggests clinic-based protocols to make the implementation of alcohol screening efficient and effective. (pp. 340–347)

## TREATMENT EFFICACY RESEARCH

Research into the efficacy of various alcoholism treatment approaches has become increasingly sophisticated in recent years. Alcohol researchers have adopted various methodological strategies that emphasize specificity and standardization of treatment, thereby facilitating the replication and comparison of different treatment approaches. Dr. Kathleen M. Carroll reviews some of these methodological features, such as the use of treatment manuals to guide psychotherapy and provider training to ensure correct and consistent treatment delivery. Dr. Carroll also provides various examples of how these “cutting-edge” research methods can be applied in clinical practice, allowing researchers and clinicians to better meet the needs of their patients and the challenges posed by managed care. (pp. 352–359)

